# Lossy DICOM conversion may affect AI performance

**DOI:** 10.1038/s41598-025-02851-w

**Published:** 2025-07-08

**Authors:** Robin Sebastian Mayer, Fabian Fliedner, Ingvild Frøberg Mathisen, Anna Laib, Julia Bein, Marco Eichelberg, Peter J. Wild, Nadine Flinner

**Affiliations:** 1 Dr. Senckenberg Institutes of Pathology and Human Genetics, Goethe-University Frankfurt, University Hospital Frankfurt, Frankfurt am Main, Germany; 2 R&D Department Health, OFFIS-Institute for Information Technology, Oldenburg, Germany; 3 Frankfurt Cancer Institute (FCI), Goethe University Frankfurt, Frankfurt am Main, Germany; 4 Frankfurt Institute for Advanced Studies (FIAS), Frankfurt am Main, Germany; 5 University Cancer Center (UCT) Frankfurt-Marburg, Frankfurt am Main, Germany

**Keywords:** DICOM, Image compression, Digital pathology, Computational pathology, Adversarial example, Image processing, Machine learning, Pathology

## Abstract

Many pathologies have started to digitize their glass slides. To ensure long term accessibility, it is desirable to store them in the DICOM format. Currently, many scanners initially store the images in vendor-specific formats and only provide DICOM converters, with only a few producing DICOM directly. However, such a conversion is not lossless for all vendors, and in the case of MRXS files even overlapping tile handling differs. The resulting consequences have not yet been investigated. We converted MRXS files depicting bladder, ovarian and prostate tissue into DICOM images using the 3D Histech/Sysmex converter and an open-source tool both using baseline JPEG for the re-compression. After conversion no human perceptible differences were present between the images, nevertheless they were not identical and had structure similarity indices (SSIM) of ~ 0.85 to ~ 0.96 on average, while the vendor specific converter in general achieved higher values. AI models based on CNNs and current foundation models could distinguish between the original and the converted images in most cases with an accuracy of up to 99.5%. And already trained AI models showed significant performance differences between the image formats in five out of 64 scenarios, mainly when only little data was used during AI training. So, if DICOM images are intended for a diagnostic use, all processes and algorithms must be (re-)evaluated with the converted files, as images are not identical. Nevertheless, the DICOM format is an excellent opportunity to ensure interoperability in future, as some first AI trainings with converted files did not result in systematically decreased performances.

**Supplementary Information:**

The online version contains supplementary material available at 10.1038/s41598-025-02851-w.

## Introduction

The digitization of pathology is a growing desire of many practitioners around the world hoping to enable new workflows such as remote diagnostics, collaboration with specialists, and integration of advanced algorithms and artificial intelligence (AI). Digitization has the potential to improve diagnostic accuracy, speed up processes, reduce costs and inter-observer variability, and thus enhance patient care^1–3^ .

In Pathology, one of the most important steps in digitization is the scanning of histopathological glass slides into high-resolution images, so called whole slide images (WSIs). However, most whole slide scanners produce proprietary file formats that don’t meet the FAIR (findable, accessible, interoperable and reusable) criteria^4^. Proprietary image files can only be viewed and analyzed easily with proprietary software, which limits interoperability^5^. While open-source software such as OpenSlide^6^ allows researchers to access pixel data, it is not certified as a medical device and there is no guarantee of maintenance or future compatibility.

All images that are produced nowadays during research projects and diagnostics are a valuable resource for further research in AI. Robust performance on real-world data requires huge training datasets^7^ and, therefore, data must be accessible, interoperable, readable and reusable. So, images scanned nowadays should be stored in an open, interoperable file format to make them accessible in the future. DICOM (Digital Imaging and Communications in Medicine) is the standard format for storing diagnostic images in radiology and allows multimodal patient data integration, including images and related metadata^8^. It also describes rules to store anonymized image data to help being in line with patient data protection regulations. However, pathology images differ from those in radiology. Images are stored in a tiled pyramidal format with different resolutions to ensure fast access^8^. Therefore, DICOM extensions for pathology, called supplement 122 (“Specimen Module and Revised Pathology SOP Classes”) and 145 (“Whole Slide Microscopic Image IOD and SOP Classes”), were published in 2008 and 2010, making it the most promising standard^8,9^.

The importance of DICOM as a promising standard is also highlighted in a recent survey by the Swiss digital pathology consortium, where 96% of the respondents stressed the importance of open formats to integrate scanning with Laboratory Information System (LIS) and Image Management Systems (IMS) and were in favor of avoiding vendor specific software. Furthermore, most of the respondents (80%) emphasized the necessity of interoperability and long-term sustainability^10^.

The ideal workflow is to scan tissue sections natively into DICOM, but currently most scanners do not support this.

For non-DICOM scanners and already scanned slides, converting to DICOM is the only way to ensure interoperability and long-term usability. Nowadays, most scanner manufacturers offer conversion tools (e.g. *SlideMaster* from Sysmex/3DHistech), in addition open-source tools (e.g. *wsidicomizer*^11^) exist. Some image formats (e.g. svs or ndpi) can be converted losslessly without changing the numeric values of the compressed pixel data^11^. Other formats, however, require changing the pixel data due to different tile organizations or compression schemes. In these cases, decompression and recompression is necessary, which results in modified pixel values and potential loss of information, even when using the same compression parameters^12^, because compression algorithms reduce file size by eliminating high-frequency features, which at high compression rates results in image distortion and blur^13^.

Although initial reports suggest no perceptible difference between the original image and the recompressed one^14^, downstream processes may be affected. In machine learning, small changes in pixel data, not notable for humans, like small amounts of noise, can lead to severe misclassifications, also known as adversarial examples^15^. This raises the question of whether the information loss caused by recompression is a significant issue. It is, therefore, imperative to assess the performance of AI algorithms on DICOM - converted images to ascertain whether the alteration in information is acceptable. It is important to note that the converted images can only be used in a diagnostic setting if all regulatory requirements^16^ are met post-conversion and that is important to ensure that the overall performance of the algorithms remains unchanged.

To address these challenges, we investigated the impact of DICOM conversion on MRXS (Mirax Scan) images (3DHistech/Sysmex). Our study examined both a direct image comparison as well as the performance of AI models trained with non DICOM data, including ResNet18 and current foundation models. Here we evaluated how far extracted image features are impacted and if so, whether this leads to changed and potentially wrong predictions. In addition, we also performed some first tests to investigate whether the use of repeatedly lossy compressed DICOM images (with similar file size) leads to significantly worse AI training results.

## Materials and methods

### Datasets

Ovarian datasets contain 72 WSIs from the TCGA-OV^16^ and 38 WSIs from the SIP (Dr. Senckenbergische Institute für Pathologie und Humangenetik), scanned with the PanoramicScanII (version: 2.1.1.100094 & 3.0.1.123298). For bladder tissue, 107 WSIs from the TCGA-MIBC^17^ and 17 WSIs from our institute, scanned with the PanoramicScanII (version: 3.0.1.123298), were used. Prostate tissue data included 40 slides from SIP scanned with the P1000 (version: 2.2.1.179491). All slides from TCGA were provided in Leica Aperio SVS (ScanScope Virtual Slide) format and were nominally at 40x magnification. Similarly, local SIP data were scanned at 40x magnification and were provided in MRXS format. For ovarian and bladder datasets annotations of cancerous and healthy tissue areas are available from former investigations^18,19^.

Tissue samples from our institute (SIP) were provided via the University Cancer Center Frankfurt (UCT). Informed consent was obtained from all patients before operation and the study was approved by the Institutional Review Boards of the UCT and the Ethical Committee at the University Hospital Frankfurt (project-number: UCT-5-2021). All research activities are in agreement with the relevant guidelines and regulations at our institute and the Declaration of Helsinki.

### DICOM conversion and tile extraction

All local MRXS slides (which were initially compressed after scanning with JPEG (ISO_10918_1) and Q = 80) were converted to the DICOM format using the Sysmex/3DHistech *SlideMaster* software (version: 2.7.0.206362) where no parameters for the compression algorithm could be selected by the user. *SlideMaster* uses JPEG (ISO_10918_1) with Q = 80 for image compression which leads to an average file size reduction of ~ 370mb. In addition, a second DICOM dataset was created by converting MRXS slides using the open-source tool *wsidicomizer* (version 0.10.2^11^), with default parameters (JPEG (ISO_10918_1) compression with Q = 90 and chroma subsampling = 4:2:0) leading to an average file size increase of ~ 250mb. In addition, a third DICOM dataset was created via *wsidicomizer*, which however was not used for AI experiments, with the settings that are used by *SlideMaster* (JPEG (ISO_10918_1) compression with Q = 80 and chroma subsampling = 4:2:2) leading to an average file size reduction of ~ 210mb. For bladder and prostate tissue 299 × 299 pixel tiles and for ovarian 512 × 512 pixel tiles were extracted at level 0 (~ 0.25 mpp (microns per pixel)) and level 2 (~ 1 mpp). In total 40 tiles were randomly sampled per WSI, if an annotation was available, 20 tiles per class were chosen. OpenSlide (version 1.4.1^6^) was used to access MRXS images and the *SlideMaster* generated DICOM images, while WSIDicom (version 0.10.0^20^), was used for the *wsidicomizer* DICOM images. As the original MRXS files contain overlapping tiles, OpenSlide performs an own rendering into non-overlapping tiles during accession of the pixel information. In contrast DICOM files do only contain non-overlapping tiles, making the step of an extra rendering unnecessary. Tiles from all three files were each extracted at the identical position and depict the same tissue area. The structural similarity indices (SSIM) were determined between OpenSlide extracted tiles from MRXS images and their respective counterparts from the three DICOM datasets, using the scikit-image *structural_similarity* function (version 0.24.0^21^) via multichannel analysis. For all subsequent AI experiments, only the *wsidicomizer* dataset that was created with default parameters was utilized.

### AI models

For all AI experiments, an ImageNet pretrained convolutional neural network (CNN: ResNet18) and three foundation models (cTransPath^22^, Virchow^7^, and Virchow2^23^) were used. Foundation models were used as feature extractors, while the complete ResNet18 architecture was trained. For all trainings the timm library (version 1.0.9^24^), which is based on pyTorch (version 1.13.0^25^), was used. All models used a custom head with respective features size as input, a dropout layer (0.5) and a classification layer. CrossEntropyLoss and the Adamax optimizer (lr = 0.001) were used. All models were trained for 20 epochs and to ensure robustness of the results, the training process was repeated 50 times with different patient stratified train-validation-test splits.

### Scenarios for the file type dependent performance test

When training models to differentiate between MRXS and converted DICOM images 17 SIP-based slides were used from all tissues (training:15, holdout-test:2).

For the carcinoma detection in bladder, models were trained using a large (all 107 slides) and a small (17 slides) dataset, randomly sampled in each run from the full dataset. For the ovarian dataset the same was done and in addition a tissue source site (referred as A and B) exclusive training was performed (large: all 72, site A 35 and site B 37 WSIs; small: all 17, site A 17 and site B 17 WSIs). As we have two resolutions and four architecture this results in 64 tested scenarios ((2 bladder cancer datasets + 6 ovarian cancer datasets)*2 resolutions *4 models = 64).

### Scenarios for the file type dependent AI training

Models were always trained on SIP data (original MRXS or converted DICOM) and tested on TCGA data. For each tissue, all available SIP-based slides were used (bladder: 17, ovar:38). For the ovarian dataset training was performed additionally with a small dataset size (17 WSIs). As we have two resolutions and four architecture this results in 24 tested scenarios ((1 bladder cancer datasets + 2 ovarian cancer datasets)*2 resolutions *4 models = 24).

### Statistical tests

Statistical tests were used from the scipy library (version 1.11.4) and conducted to determine if observed performance differences were significant. If all necessary assumptions were met, the appropriate statistical method (ANOVA) was utilized. Otherwise, a non-parametric method (Kruskal-Wallis) was used. Finally, p-values were corrected via the Bonferroni correction to account for multiple comparisons. After that, pairwise significance was determined via an appropriate test (Tukey’s HSD / Mann-Whitney U-test).

## Results

### Images are indistinguishable for the human eye, but not identical

To evaluate the effect of DICOM conversion on image quality for already existing MRXS files we used two different converters: (1) *SlideMaster*, which is the vendor specific converter from 3DHistech/Sysmex and (2) the open source *wsidicomizer* tool, which for MRXS files makes use of the OpenSlide library to retrieve image data from the original file. Tissue from three organs was digitized with different scanners and used as input for the converters: Ovarian cancer tissue and bladder cancer tissue (both Panoramic Scan II) and prostate cancer tissue (P1000).

**Fig. 1:**
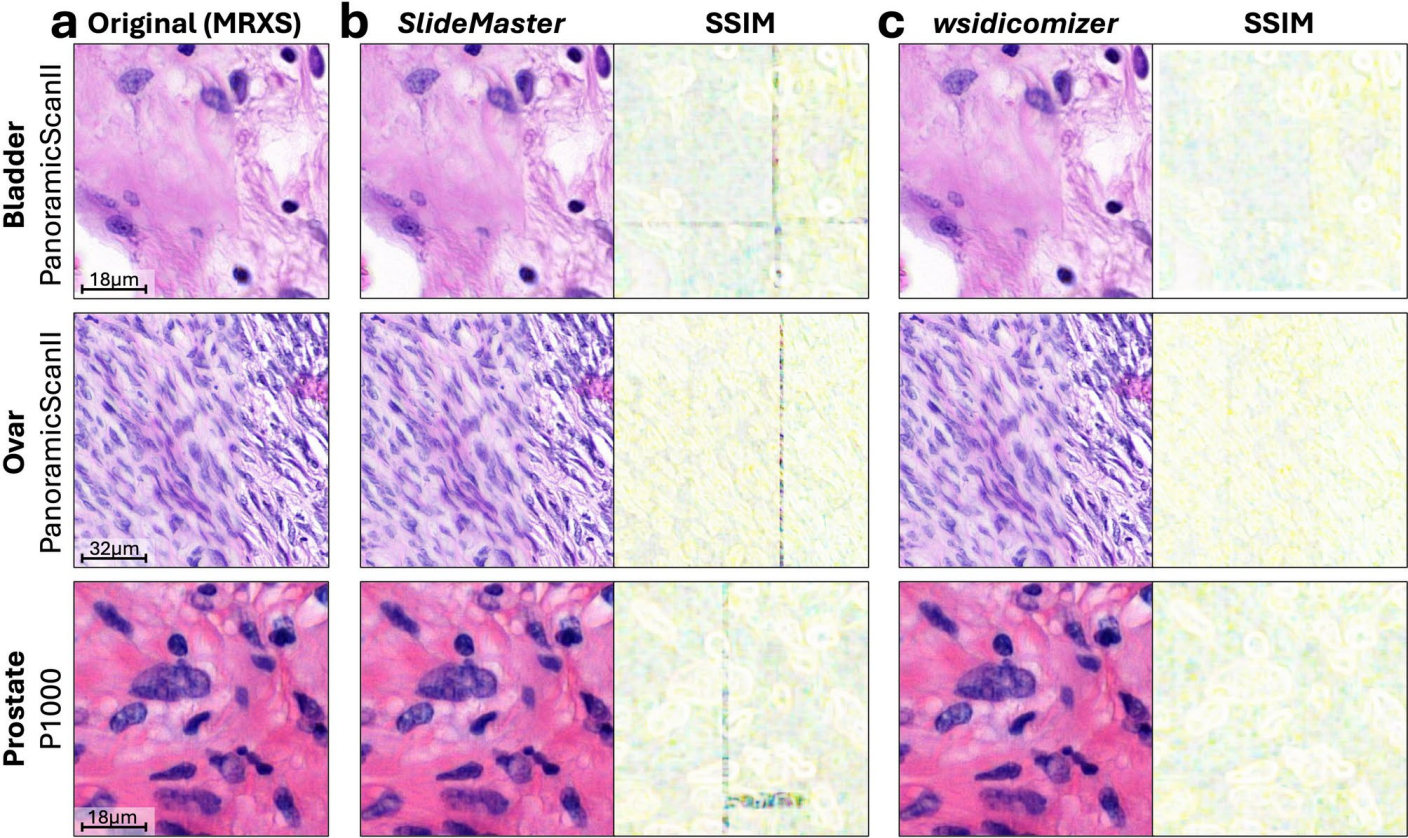
Qualitative and structural differences between original and DICOM-converted images. Tiles were extracted from the original MRXS WSI (**a**), the *SlideMaster* converted DICOM WSI (**b**) and the *wsidicomizer* converted DICOM WSI (**c**) at 0.25 mpp from WSIs of bladder, ovarian, and prostate tissue from a local SIP cohort. For (**b**) and (**c**) structural similarity index (SSIM) is shown between the original MRXS and the converted file, too.

No discernible differences were found between original and DICOM-converted tiles by visual inspection (Fig. 1) by a pathologist and several scientists displaying the extracted PNG tiles simultaneously and next to each other on the same monitor. The same is true for lower-resolution tiles (1.0 mpp; Fig. S1). But an 8 × 8 pixel area zoom-in showed small variations in pixel values (Fig. S2). In MRXS files individual tiles are stored in an overlapping manner, which need to be stitched together during viewing. As most DICOM WSI viewers expect non-overlapping tiles, stitching and retiling is necessary before conversion of MRXS files. Stitching artefacts are present in the MRXS file accessed via OpenSlide and in the *wsidicomizer* converted DICOM, which also uses OpenSlide to retrieve the original data and are absent in the *SlideMaster* generated DICOM file (Fig. S3). As stitching artefacts are also absent when the MRXS file is viewed with the proprietary software, this indicates that the presence of stitching artefacts depend on the reader to access original pixel data in the MRXS file and not the converter itself. Although these discrepancies are not readily discernible through qualitative observation, they might become more evident through quantitative analyses, such as the use of metrics like the Structural Similarity Index (SSIM).

**Fig. 2:**
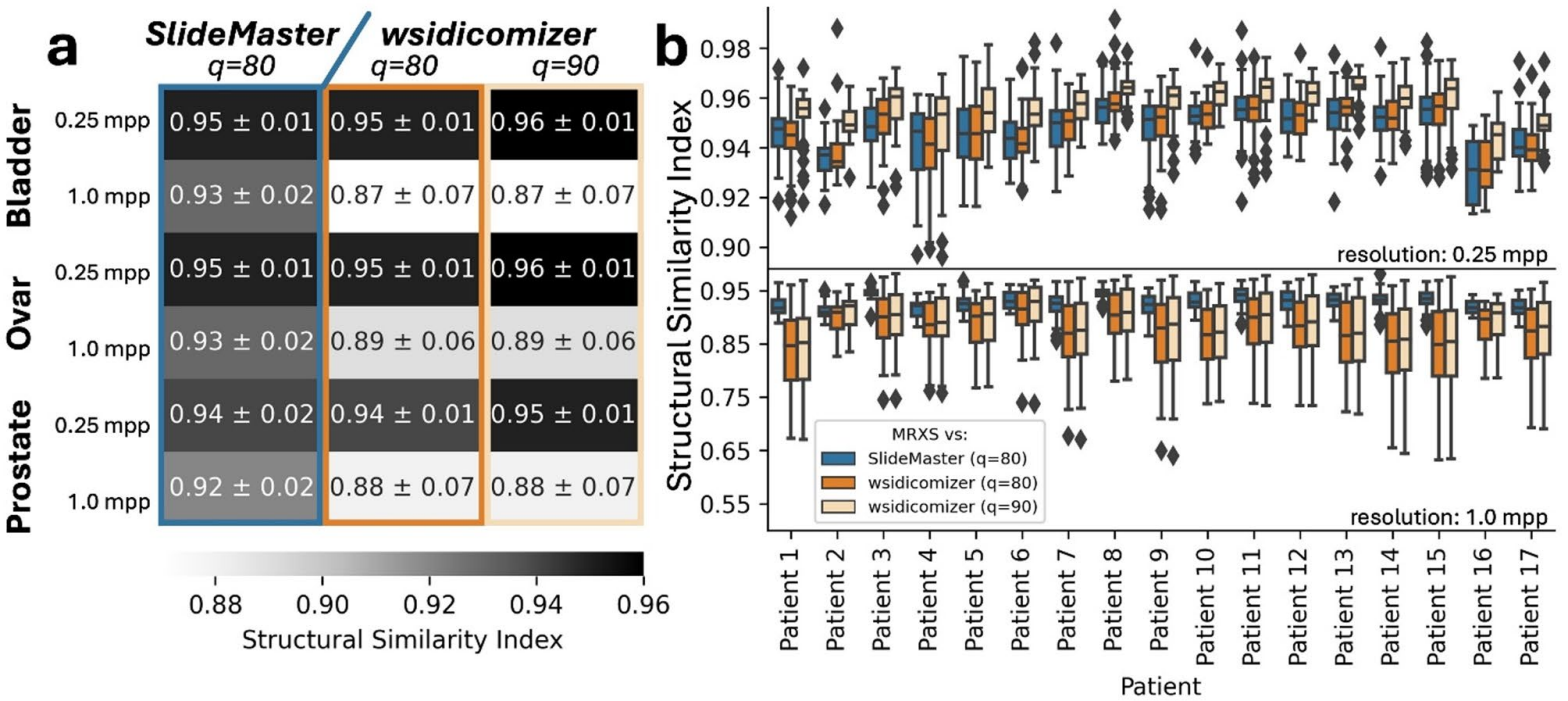
Quantitative differences between original and DICOM-converted images. Structural similarity index (SSIM) was calculated between original and DICOM-converted tiles extracted from WSIs of bladder, ovarian, and prostate tissue using two conversion tools: *SlideMaster* (blue) and *wsidicomizer* (*SlideMaster* settings (q = 80): orange, default settings (q = 90): beige). (**a**) Heatmap depicting the dataset based SSIM values against original MRXS tiles, given numbers are mean and standard deviation. (**b**) Boxplot depicting the patient-based variation between tiles for bladder tissue tiles at 0.25 mpp (top) and at 1.0 mpp (bottom).

Calculating and visualizing the SSIM for the given examples revealed structural differences between original and converted images: In line with our previous findings, these differences were most evident along the initial stitching lines comparing OpenSlide accessed MRXS tiles (using the OpenSlide stitching during tile extraction) and *SlideMaster* converted tiles (using the vendors own API for stitching during file conversion) (Fig. 1B). For the *wsidicomizer* and the MRXS-OpenSlide tile only minor differences along the initial stitching lines appear (Figs. 1C and 2A), as both are using OpenSlide which introduces more stitching artefacts than the proprietary software.

Average SSIM values between the datasets show that the measured similarity tended to be higher for *SlideMaster* than for *wsidicomizer* in 1 mpp resolution across all examined tissues. For higher resolution (0.25mpp) average similarities are more alike between the tools, giving a slight edge to *wsidicomizer* with default settings (Q90; chroma subsampling: 4:2:0). Using the *wsidicomizer* with the settings used by *SlideMaster* (Q80; chroma subsampling: 4:2:2) results in more similar SSIM values compared to *SlideMaster.* In short, the structural differences between original and DICOM converted images were generally higher in low magnifications (1.0 mpp) compared to higher ones (0.25 mpp; Fig. 2A, Fig. S4). For the lower magnification *SlideMaster* also achieved higher SSIM values in all tested tissues, independently of the compression parameters used for *wsidicomizer*.

Further, we can observe that the similarity, while seemingly constant for the *SlideMaster* generated DICOM files, can vary greatly across different slides within one dataset for the *wsidicomizer* generated DICOM files, especially at 1 mpp resolution, irrespectively of the used compression settings (Fig. 2B). This indicates a different strategy to generate the lower resolution tiles between *SlideMaster* and the *wsidicomizer*. These slide-by-slide differences can be seen in the 1 mpp bladder tissue dataset where the lowest average SSIM observed for *wsidicomizer* tiles (default setting: Q90; chroma subsampling: 4:2:0) was for patient 1, with an average SSIM of ~ 0.84 and the highest value of ~ 0.92 for patient 6. However, even with such low SSIM, barely a difference between the differently converted images is apparent by naked eye (Figure S5). Similarly, varying effects by patient could also be observed in other tissues, as well as other resolutions (Figure S6) indicating a general slide-by-slide difference of DICOM conversion impact.

To conclude, images are indistinguishable between original and DICOM-converted ones for human observers when viewed in a resolution relevant for diagnosis. Nevertheless, quantitative discrepancies are observable and can exhibit considerable variability within one dataset. This emphasizes the necessity for further investigation into the potential impact on downstream analyses.

### Original and converted images could be differentiated by AI

**Fig. 3:**
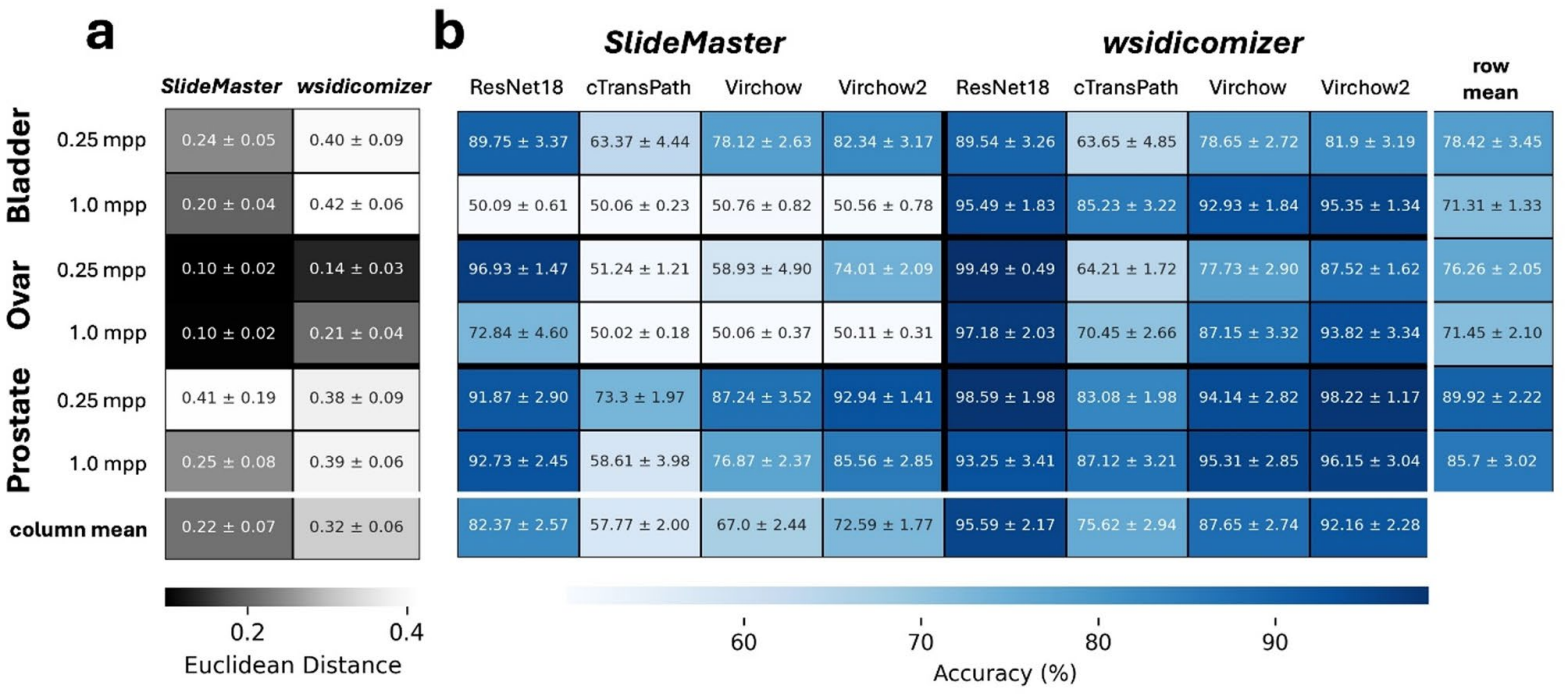
AI models detect differences between original and DICOM converted images. (**a**) The heatmap visualizes the average Euclidean distance (± standard deviation) between feature vectors, which were extracted with cTransPath from original and converted tiles. (**b**) The heatmap shows the accuracy of AI models in distinguishing original tiles from DICOM-converted ones (± standard deviation). Models (ResNet18, cTransPath, Virchow, and Virchow2) were trained for each tissue-resolution combination (*n* = 50), with patient-stratified splits. Means across all tissues/resolutions are averaged at the bottom of the heatmap, while means across all models per tissue-resolution case are shown on the right.

As machine learning and AI will become more common in pathology, it is important to determine whether these small discrepancies could affect the results of AI analyses. Therefore, we first investigated whether the differences in the images are also reflected in features extracted from foundation models. We used three foundation models (cTransPath, Virchow, and Virchow2) to extract features from image tiles and calculate the Euclidean distance between the OpenSlide extracted MRXS original and DICOM converted features (Fig. 3A). Only marginal differences were observed, with average values between 0.10 and 0.42 for *SlideMaster* and *wsidicomizer*-generated tiles. For orientation, the Euclidian distance between feature vectors of different tiles from one WSI (Bladder, 0.25mpp) was 3.5 ± 0.5. In agreement with the SSIM values, features from *SlideMaster*-extracted tiles tend to have a smaller Euclidian distance to the original tile features even in cases where SSIM was higher in wsidicomizer-tiles. In addition, lower SSIM values did correlate with a higher Euclidean distance between features, which indicates that AI models could potentially pick these differences up. For *SlideMaster* correlation varies between − 0.03 and − 0.48 and is in all but one dataset (Bladder 0.25mpp) lower compared to the *wsidicomizer* where the correlation lies between − 0.02 and − 0.75 (Figure S7).

It is of great interest to ascertain whether the difference in the features that were introduced by the DICOM conversion could be employed to differentiate between the original MRXS-based tiles and those derived from DICOM files. To this end, a ResNet18 and three foundation models were trained, with the objective of differentiating between the original tiles and those converted by *SlideMaster* and *wsidicomizer*. Here we observed that the distinguishing capability depends on the tissue type (aka scanner), resolution, conversion software and finally also the model used (Fig. 3B). The differentiation of tiles in all three datasets was possible with at least one of the tested models. However, for the bladder and ovarian cancer tiles, the foundation models were unable to discriminate between original and *SlideMaster* converted image tiles in low resolution (1.0 mpp) where ResNet18 succeeded only in ovary. *Wsidicomizer* generated tiles were generally easier to identify by the models (+ 20% average) and tiles with a higher resolution were easier to differentiate, too. In general, performances were highest for ResNet18 followed by Virchow2, Virchow and cTransPath. This suggests that the cTransPath representation is less affected by conversion differences and has fewer conversion-specific features, potentially making it more robust against the induced changes in image quality. Furthermore, it is not surprising that ResNet18 was best suited to differentiate both types as, in contrast to the foundation models, their feature extraction was trained to differentiate both image types in this experimental setup. When inspecting the correctly and wrongly predicted tiles, only minor difference between SSIMs could be determined (Figure S8), indicating that quantity of changes present in the image did not determine prediction success for the models. One possible strategy of the ResNet18 models is to focus on blurry areas while identifying DICOM images: When observing GradCAMs for some ResNet18 predictions sharper areas were given greater scrutiny for the classification of an original MRXS tile (Fig. S9). This is in accordance with the established fact that compression algorithms do eliminate fine structures.

Taken together, current foundation models extract features that depend on the compression status and overlapping tile handling, and are characteristic for the MRXS and DICOM converted slide. Otherwise, differentiation between the two would be impossible. This is an undesired characteristic as changes in the feature distribution between training and test datasets result in a domain shift and may lead to an undesired behavior of AI models.

### Significant performance differences for existing AI models between original and converted tiles are rare but exist

**Fig. 4:**
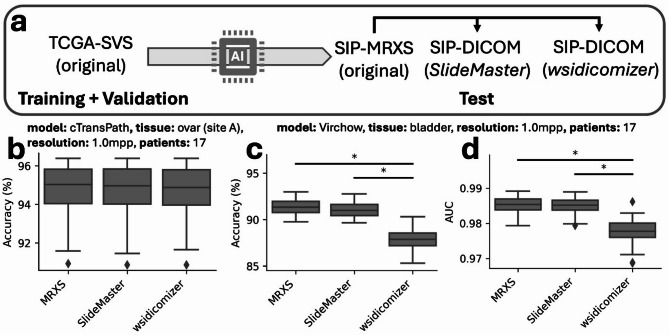
Significant performance differences between OpenSlide extracted original MRXS and DICOM converted images exist during AI model inference. (**a**) AI models (ResNet18, cTransPath, Virchow, and Virchow2) were trained on TCGA data and tested on local SIP data for the OpenSlide extracted original MRXS and DICOM converted images for different resolutions and data set sizes. Exemplary results are shown in (**b–d**). Results in (**b**) belong to the cTransPath model trained with a small subset (*n* = 17) from site A (ovarian cancer) at a resolution of 1 mpp. Results in (**c**,**d**) belong to the Virchow model trained with a small subset from Site A and B (*n* = 17) from bladder data at a resolution of 1 mpp.

Given the potential for domain shift, it is imperative to ascertain whether down-stream prediction tasks are indeed impacted. Therefore, we trained models on TCGA data of bladder or ovarian tissue to classify tiles as healthy or cancerous and subsequently evaluated the models on SIP OpenSlide extracted MRXS data, as well as the corresponding DICOM-converted tiles (Fig. 4A). Trainings were performed for different resolutions (0.25 and 1.0 mpp) and different data subsets, to simulate small and monocentric datasets, leading to 64 training scenarios.

From these, we can observe that usually predictions after DICOM conversion are unchanged. For example, accuracy showed hardly any differences for the ResNet18 (training data: ovary, small subset with 17 WSIs, site A) differentiating cancerous and non-cancerous tiles at 1.0 mpp (Fig. 4B), which is exemplary for most of the tested scenarios (59 of 64; Table S1). However, in five cases a statistically significant difference was observed in AUC or accuracy, where one scenario shows a difference in accuracy and AUC (Bonferroni corrected p-value < 0.05). Here the *wsidicomizer* conversion led to a lower performance of a Virchow-based model trained on a small bladder cancer dataset at 1.0 mpp, which exhibited an approximate 4% decline in accuracy and a 0.07 reduction in AUC (Fig. 4C,D). On the other hand, also significant positive effects of the conversion are observable for both tools either in AUC or accuracy (Figure S10, Table S1).

In general, the performance differences, even when statistically significant, were minimal. We can, therefore, conclude that the utilization of AI models on DICOM-converted tiles derived from MRXS is in general possible. However, the presence of statistically significant performance changes, even though rare, must lead to caution and a re-evaluation of the models for the specific use-case when a diagnostic use is intended.

### DICOM converted images are not systematically worse for AI training

**Fig. 5:**
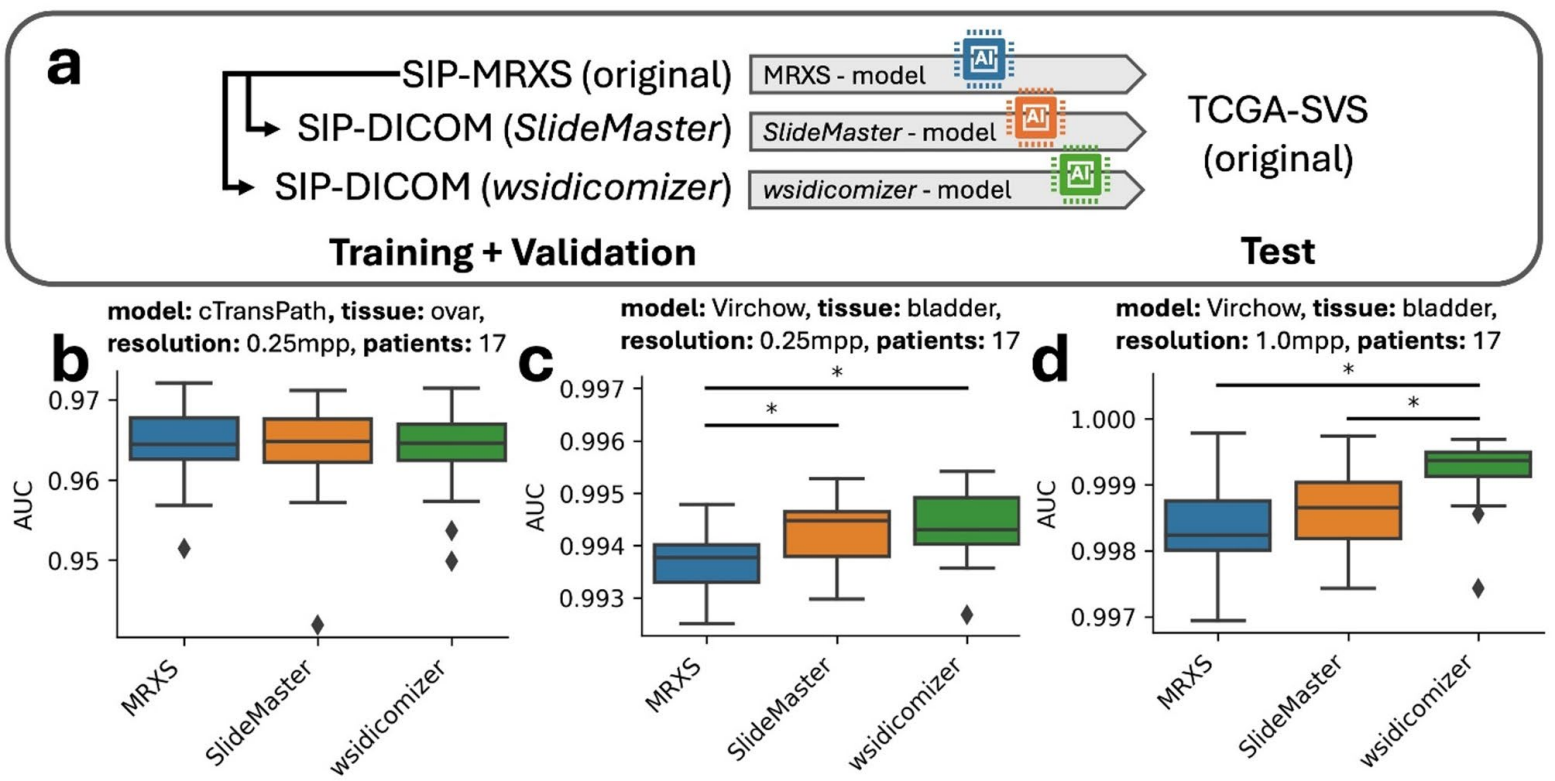
AI training with OpenSlide extracted original MRXS and DICOM converted images does not result in systematic performance improvements or reductions. (**a**) AI models (ResNet18, cTransPath, Virchow, and Virchow2) were trained on local SIP data with the original MRXS (OpenSlide extracted) or the DICOM converted images and tested on TCGA data for different resolutions and data set sizes. Exemplary results for the AUC are shown in (**b–d**). Results in (**b**) belong to the cTransPath model trained on ovarian data at a resolution of 0.25 mpp and set of 17 patients. Results in (**c**,**d**) belong to the Virchov model trained bladder data at a resolution of 0.25 and 1 mpp and a set of 17 patients.

Finally, the potential impact of training AI models with DICOM-converted images in comparison to OpenSlide extracted original images was examined, to check the suitability of DICOM converted files for future studies. Models were trained exclusively on tiles derived from the OpenSlide extracted original or their *SlideMaster*/*wsidicomizer* converted counterparts from our institute (Fig. 5A). This analysis was conducted using bladder and ovarian tissue datasets, at both 0.25 and 1.0 mpp resolution and differently sized subsets resulting in 24 scenarios.

When these models were evaluated on TCGA-derived test sets, nearly no performance differences for ovary and minimal differences were observed for bladder (2 of 24; Table S2). As an example, cTransPath models trained on ovarian tissue (0.25 mpp) AUC does not differ (Fig. 5B). Statistical significance in AUC were only present in two bladder cases. The AUC improved minorly (P-value: ~0.0005) in Virchow models trained on bladder tissue (0.25mpp) for *SlideMaster* and *wsidicomizer* tiles (Fig. 5C). Moreover, in Virchow models trained on bladder with 1.0 mpp the AUC also saw a minor but statistically significant improvement for the *wsidicomizer* ( + ~ 0.001; Fig. 5D). However, for both models there was no statistically significant difference in the accuracy after correction for multiple testing. Additionally, the performance differences did not follow a consistent trend and also a decreased performance is seen without correction for multiple testing (e.g. AUC 0.929 (MRXS-training) > 0.927 (*SlideMaster*-training) > 0.925 (*wsidicomizer*-training) for Virchow model trained on ovarian tissue (1.0mpp, small dataset; Table S2).

As performance alterations were rare and even yield minor improvements when statistically significant, training on DICOM-converted tiles generally does not impair the quality of the resulting models. This finding further supports the procedure of converting existing files to DICOM for long term storage, thereby ensuring their suitability for further research in the future.

## Discussion

In this article we provide a detailed insight into the image quality after DICOM conversion (Figs. 1 and 2). No differences that are visible for humans were found between MRXS and its DICOM counterpart at the resolutions used in diagnostics.

Since it is well known that there are no differences in the diagnosis between conventional light microscopy and digitized WSIs^26^ in general and that lossless compression of image data is not necessary^27^, we expect no influence on the routine workflow with re-compressed DICOM images. In agreement, Mori^14^ also found no visible differences between the two image types at 40x resolution, however he also used a different scanner (Hamamatsu producing ndpi files) and conversion tool (Infinitt). For ndpi files it is possible to convert images losslessly into DICOM^11^ but Infinitt also performs a lossy recompression to optimize tile sizes for viewing with their own DICOM viewer^14^. However, in his paper an analysis of how much subsequent automated image processing is influenced is missing.

We have shown that pixel data from OpenSlide extracted MRXS and DICOM converted files can be distinguished (Fig. 3), indicating that features exist that are picked up by AI models. GradCam (Figure S9) results suggest that one possible strategy of the algorithms is to focus on sharp regions while classifying tiles as MRXS and unsharp regions for DICOM (where the lossy re-compression has potentially led to the blurring of fine image features). So, the presence of features that enable the differentiation of both file types introduces the risk of a domain shift, if only images that have been compressed once are used exclusively in AI training. Furthermore, our screening approach using identical image regions from each file (Fig. 4) revealed the possibility of significant performance differences between MRXS and the recompressed *wsidicomizer* tiles, both using the same stitching mechanism and differ only in the compression indicating that re-compression is the predominant factor. This finding is in agreement with a stress test study which has shown that AI models typically become worse with increasing amount of blur and compression^28^. Furthermore, it has been shown that out of focus regions, i.e. blurry and unsharp areas in WSIs, reduce performance of AI cancer detection^29^. The fact that the changes in our images can also result in performance differences, which in contrast to those in the aforementioned papers are virtually invisible, is of course a notable difference. But it is also reported that 95% of AI performance is maintained with a compression of up to 85%^13^, and also our change in accuracy with 3–6% is only minor. However, there is a possibility of a statistically significant difference between MRXS and DICOM-converted images. This highlights the need for careful evaluation of diagnostic AI models with appropriate test datasets^30^. Furthermore, Whole Slide Imaging (WSI) systems already operate under significant regulatory controls. In the US, the FDA classifies them as Class II medical devices requiring special controls, while under the EU’s IVDR, they typically fall into the risk Class C category for diagnostic use^31^. Complementing this, CAP (College of American Pathologists) guidelines recommend revalidation of the entire WSI system whenever modifications are made^32^ which is also reflected by our results and highlight the necessity of these regulatory frameworks and validation protocols.

Finally, we tested whether using DICOM-converted images in AI training led to worse predictions, as this would be a clear argument against converting vendor-specific files that are intended to train AI models in the future. We showed that statistically significant differences can occur but are not systematic and training based on converted DICOM images could also lead to improved results (Fig. 5, Table S2), making it safe to use converted images without hesitation as file access is guaranteed due to the DICOM format. A small reduction in image quality due to lossy compression seems unlikely to cause any practical problems in training and/or testing^33^, however combining different levels of compression in training and model application can lead to severe effects^33^.

At the end it is important to mention that we have not considered digitization noise in our study. Digitization noise also leads to the fact that one glass slide is represented differently with every digitization, which also potentially changes AI predictions. Whether the effect of noise, which underlays a random process, or the systematic changes by the DICOM compression have a bigger effect on AI predictions needs to be investigated in the future. To do this, multiple scans of the same physical glass slide or sensor calibration data is necessary to e.g. compute the standard score^34^.

Taken together, our data lead to the hypotheses that (1) stitching is a minor factor in DICOM conversion (because original and recompressed tiles could be distinguished using the same stitching procedure; Fig. 3) and that (2) recompression using the original parameters is preferable to higher quality recompression (because *wsidicomizer* Q90 tiles more often result in statistically significant performance differences compared to *SlideMaster* Q80 tiles). But to really confirm this, additional experiments on tiles excluding areas scanned in an overlapping manner and on *wsidicomizer* generated tiles using *SlideMaster* like settings are needed.

## Conclusions

The implementation of the DICOM format in pathology to store and manually evaluate WSIs presents minimal risk, even when the MRXS format was lossy converted. In comparison to the open-source tool, the vendor-specific converter *SlideMaster* introduced fewer alterations, especially for the non-base-layer resolutions. Nevertheless, caution needs to be taken when using AI-driven workflows, particularly in diagnostics. Models that have not been exposed to recompressed DICOM tiles require careful re-evaluation. To avoid performance changes, inference could be performed on the original format before conversion and storage. However, this compromises reproducibility of results if the original files are not retained. As an alternative a lossless compression with e.g. JPEG-LS (ISO 14495-1) or JPEG2000 (ISO 15444-1), which achieve compression ratios of 4:1 to 5:1^35^, could be used during DICOM conversion of already existing files to avoid the second change of pixel data and so all potential changes in AI predictions, however in this case DICOM files would increase by a factor of 3–4 in size.

Despite these challenges, the benefits of adopting DICOM outweigh the drawbacks. The format’s data integration offers new analysis opportunities. Furthermore, the interoperable nature of DICOM will enable researchers to access stored data also in the far future, while AI training performance with this data is not affected systematically. Consequently, DICOM adaptation offers great opportunities with relatively low risks for digital pathology.

## Electronic supplementary material

Below is the link to the electronic supplementary material.

Supplementary Material 1

## Data Availability

The WSI’s from the SIP are not publicly available due to legal restrictions governing patient privacy protection. They are available upon reasonable request from the corresponding author, after agreement from the institute.
