# Supplementary material for: Lossy DICOM conversion may affect AI performance
Source: Sci Rep. 2025 Jul 8;15:24470. doi: 10.1038/s41598-025-02851-w (PMC12238447; doi:10.1038/s41598-025-02851-w)
Supplement: Supplementary file 1 — Supplementary Material 1 [file 41598_2025_2851_MOESM1_ESM.pdf]

Supplementary Material for:

Lossy DICOM conversion may affect AI performance

Robin Sebastian Mayer[1], Fabian Fliedner[1], Ingvild Frøberg Mathisen[1], Anna Laib[1], Julia Bein[1], Marco Eichelberg[2], Peter J. Wild[1,3,4,5], Nadine Flinner[1,3,4,5*]

1 Goethe-University Frankfurt, University Clinic, Dr. Senckenberg Institutes of Pathology and Human Genetics, Frankfurt am Main, Germany.
2 R&D Department Health, OFFIS-Institute for Information Technology, Oldenburg, Germany.
3 Frankfurt Cancer Institute (FCI), Goethe University Frankfurt, Frankfurt am Main, Germany.
4 Frankfurt Institute for Advanced Studies (FIAS), Frankfurt am Main, Germany.
5 University Cancer Center (UCT) Frankfurt-Marburg, Frankfurt am Main, Germany
* Correspondence: nadine.flinner@ukffm.de

Table of contents

Figure S1: Qualitative and structural differences between original and DICOM-converted images.... 2

Figure S2: Pixel value differences between original and DICOM converted images.......................... 2

Figure S3: Handling of stitching artifacts by DICOM-conversion tools.. 3

Figure S4: Quantitative differences of structural similarity index of DICOM-converted images. 3

Figure S5: Example bladder tissue tile from low SSIM patient. 4

Figure S6: Additional quantitative differences between original and DICOM-converted images. 5

Figure S7: Correlation of SSIM and distance of extracted feature vectors from original and DICOM-converted images. 6

Figure S8: Predictability of tile origin based on differences in image and feature representation....... 7

Figure S9: GradCAMs for ResNet18 predicting tile origin file format in an ovar tile. 8

Figure S10: Possible performance increase between original and DICOM images exist during AI model inference. 9

Table S1: Performance of ResNet18 and Foundation Model-Based Feature Extractors for Carcinoma Detection. 11

Table S2: Comparison of performance of training on DICOM-converted images against original mrxs. 13

Detailed Methods 13

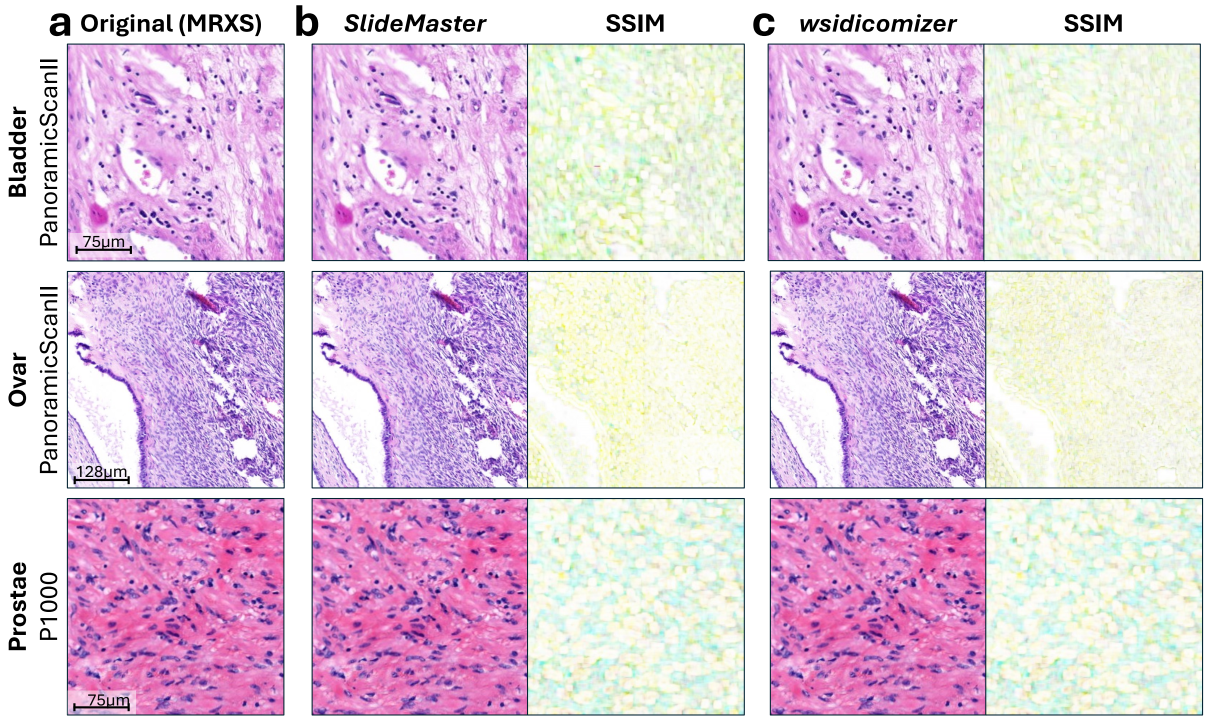

Figure S1: Qualitative and structural differences between original and DICOM-converted images. Tiles were extracted from the original MRXS WSI **(a)**, the *SlideMaster* converted DICOM WSI **(b)** and the *wsidicomizer* converted DICOM WSI **(c)** at 1.0 mpp from WSIs of bladder, ovarian, and prostate tissue from a local SIP cohort. For (b) and (c) structural similarity index (SSIM) is shown between the original MRXS and the converted file, too.

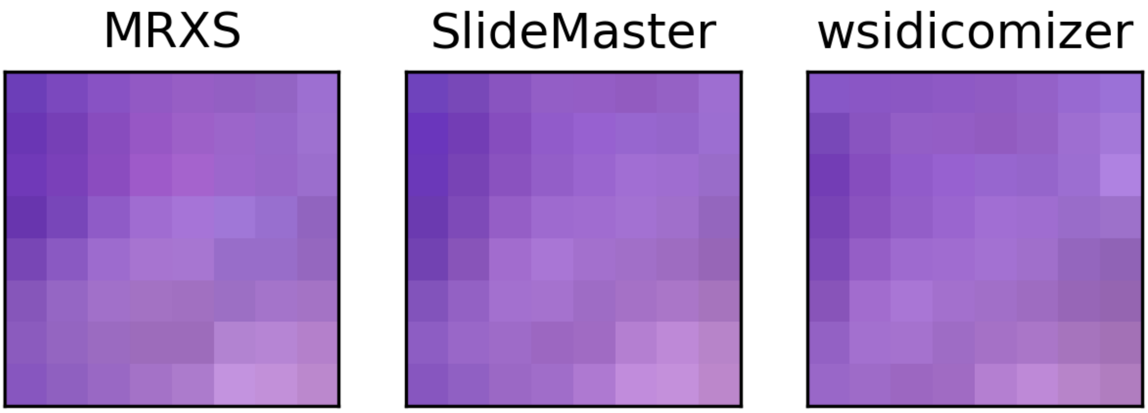

Figure S2: Pixel value differences between original and DICOM converted images. Zoomed in view of a tile from ovarian tissue at 0.25mpp. Visualized is the same 8x8 pixel area from the original MRXS, *SlideMaster* converted and *wsidicomizer* converted slide.

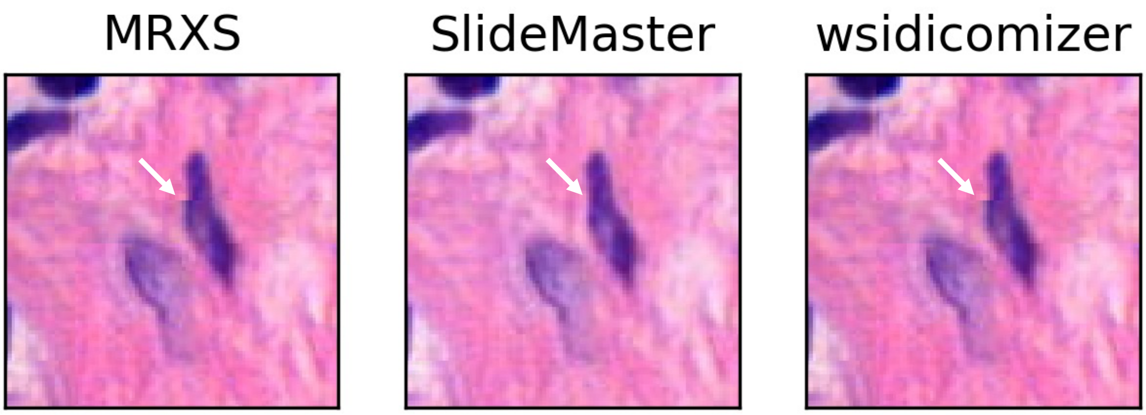

Figure S3: Handling of stitching artifacts by DICOM-conversion tools. Stitching artifacts present in the original MRXS slide are handled differently by the conversion tools *SlideMaster* which introduced a smoother transition and *wsidicomizer* which kept the original artifact. These differences are likely caused by the reader used during conversion, rather than the conversion tool itself.

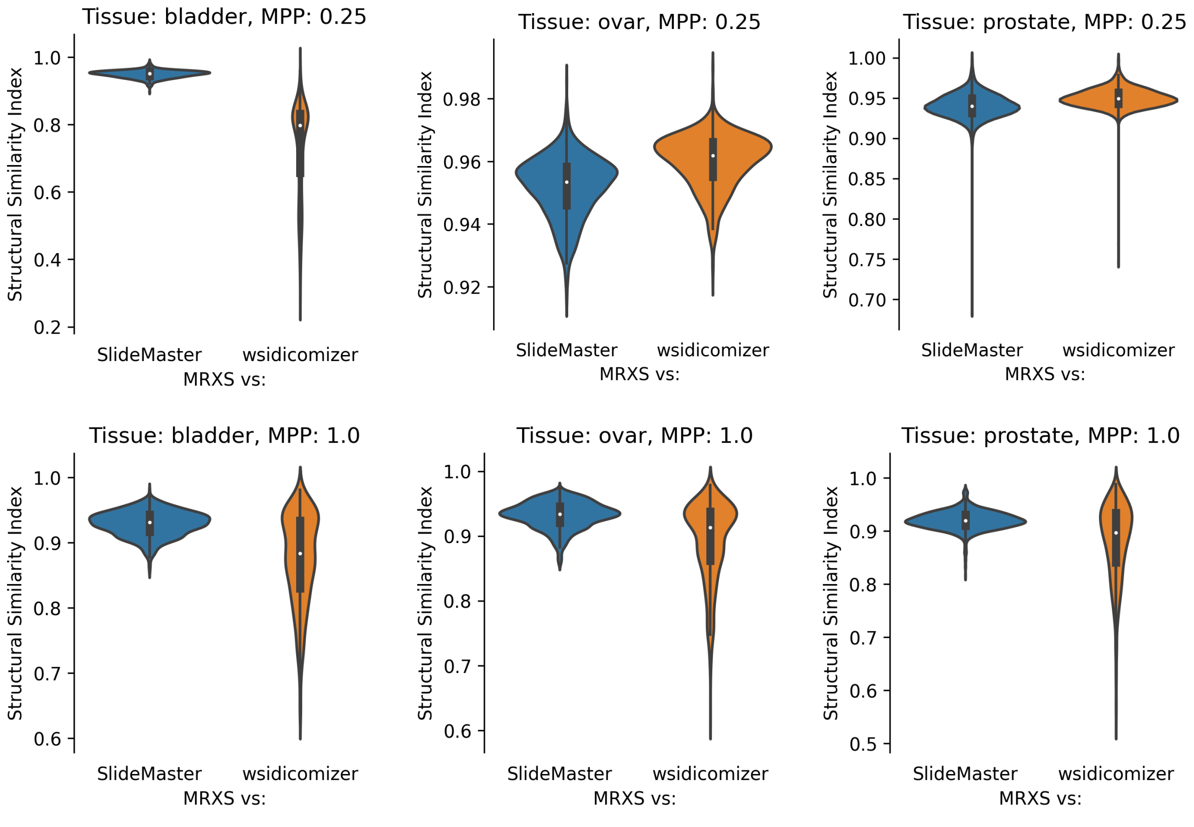

Figure S4: Quantitative differences of structural similarity index of DICOM-converted images. For slides of bladder, ovarian, and prostate tissue from SIP, the SSIM was calculated between the original MRXS data and the converted images, using either *SlideMaster* (blue) or *wsidicomizer* (orange). The calculations were performed across all tiles within the respective datasets, at both 0.25 mpp (top row) and 1.0 mpp (bottom row) resolutions.

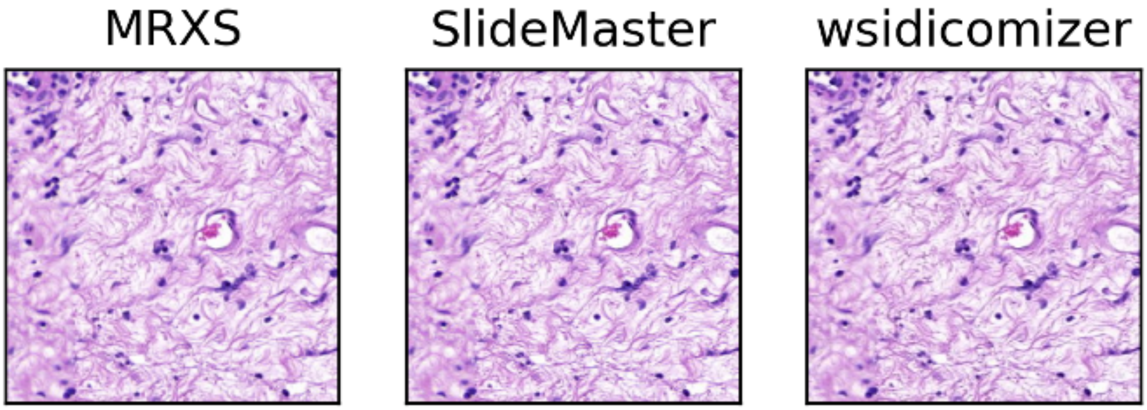

Figure S5: Example bladder tissue tile from low SSIM patient. Shown is a tile from patient 1 of the bladder tissue dataset at 0.25 mpp. Despite the low SSIM of 0.67 there are no differences observable between the original MRXS, *SlideMaster* converted and *wsidicomizer* convert tile by naked eye.

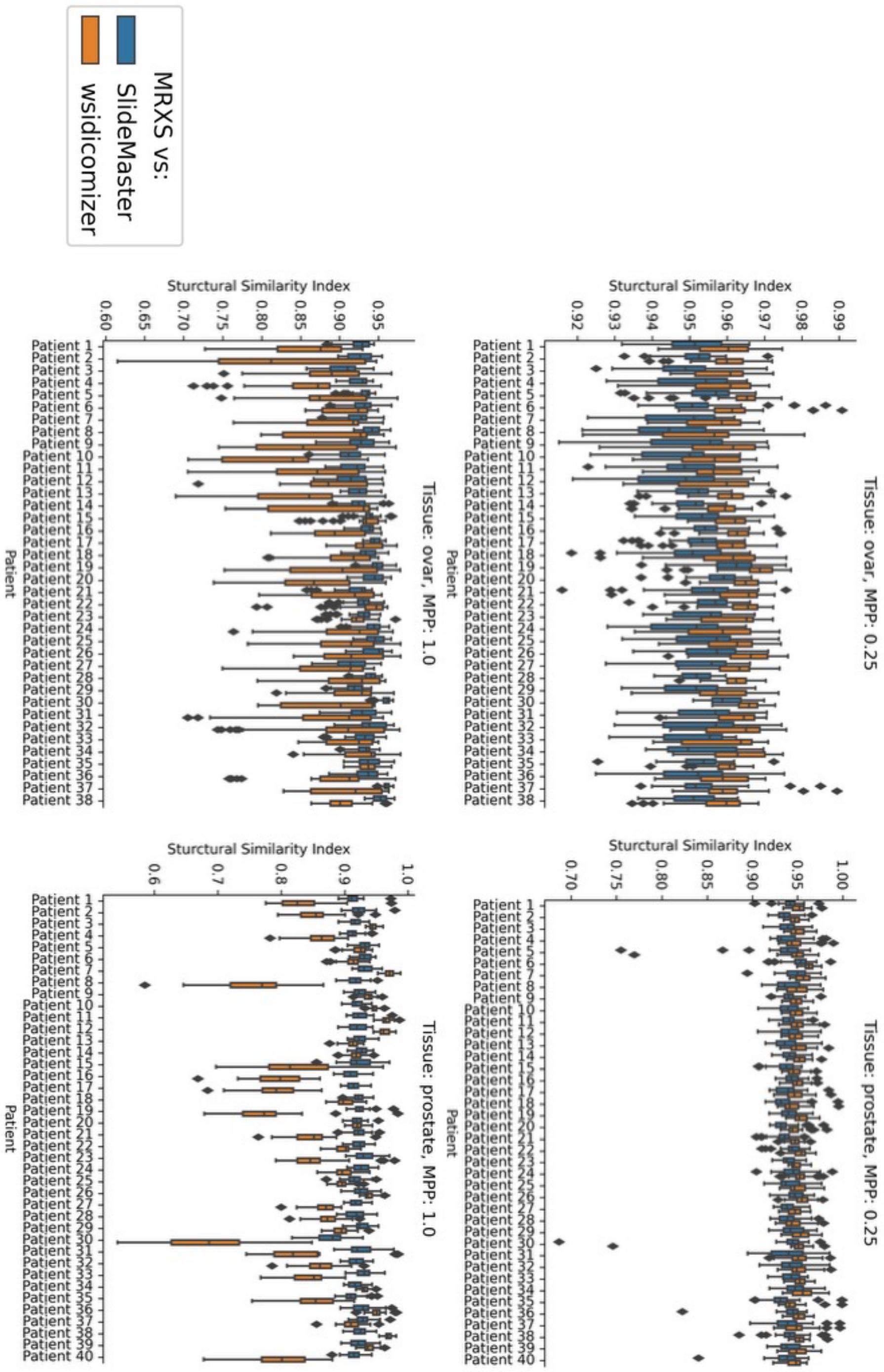

Figure S6: Additional quantitative differences between original and DICOM-converted images. Structural similarity index (SSIM) was calculated between original and DICOM-converted tiles extracted from WSIs of ovarian and prostate tissue using two conversion tools: *SlideMaster* (blue) and *wsidicomizer* (orange). Shows a boxplot depicting the patient-based variation between tiles at 0.25 mpp (top row) and 1.0 mpp (bottom row).

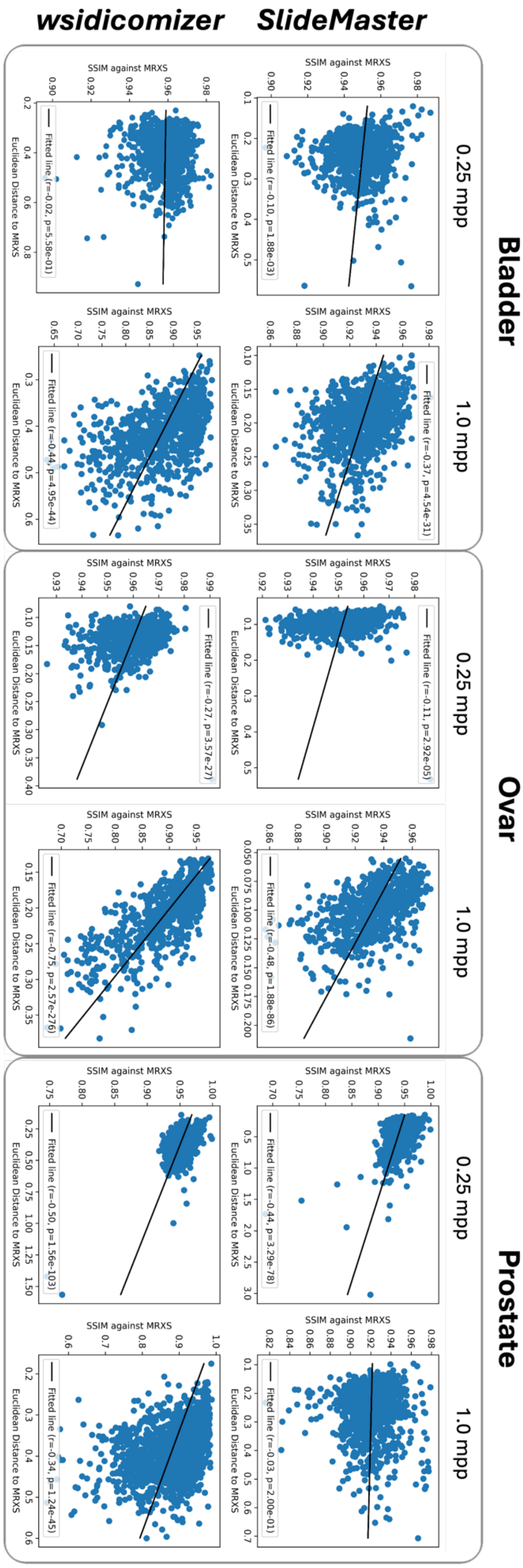

Figure S7: Correlation of SSIM and distance of extracted feature vectors from original and DICOM-converted images. For tiles of slides from bladder, ovarian and prostate tissue the SSIM between original and DICOM-converted images are calculated. Additionally features of these tiles were extracted using cTransPath and Euclidean distances between features of original MRXS and DICOM-converted images are calculated. Shown are correlation plots between the SSIM of images and distance of feature vectors.

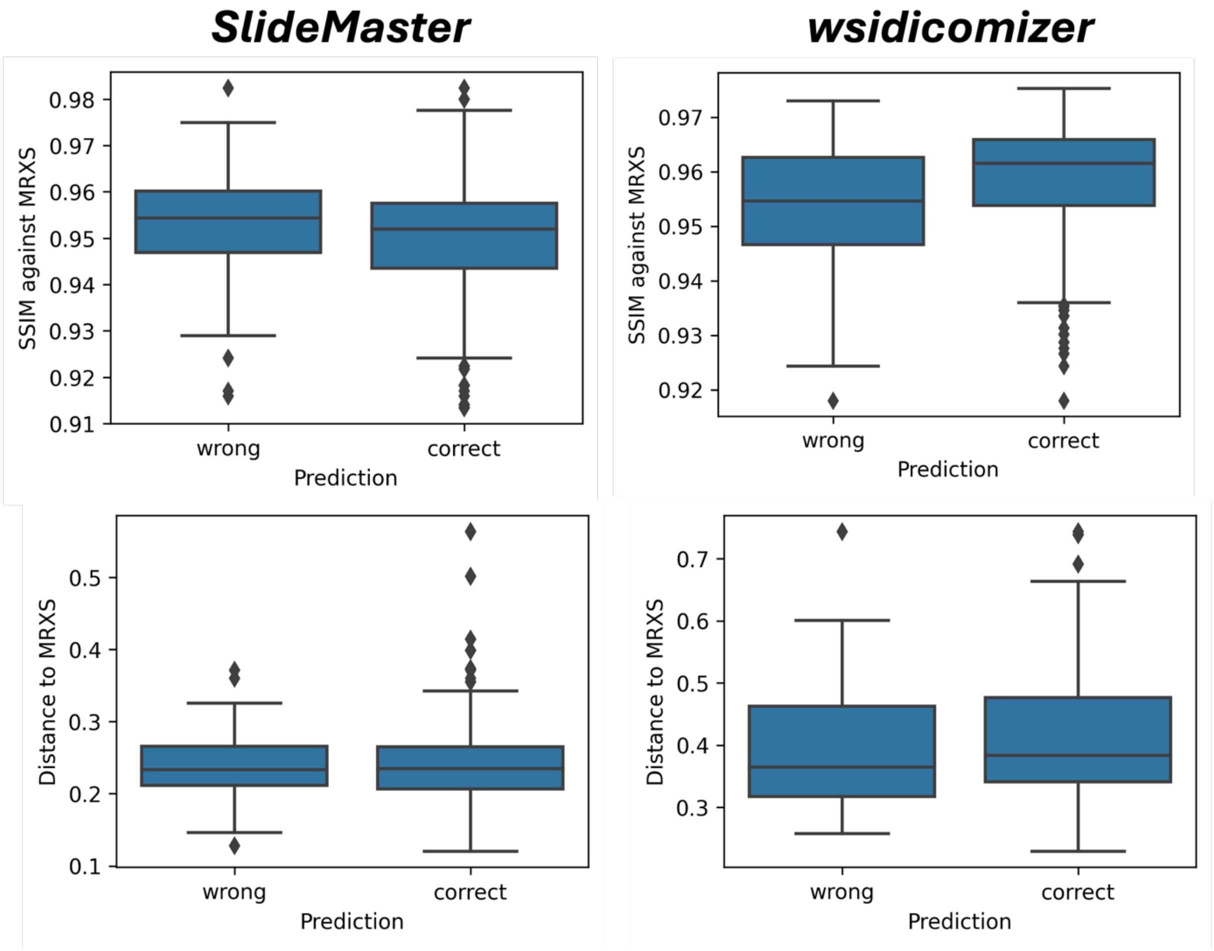

Figure S8: Predictability of tile origin based on differences in image and feature representation. ResNet18, cTransPath, Virchow and Virchow2 based models were trained to detect the origin of a tiles (original vs. DICOM converted) for two different conversion tools. Then differences of wrongly and correctly predicted tiles regarding both the structural similarity between original and converted tiles, as well as euclidean distances between extracted features were analyzed. The boxplots show the example case from ResNet18 in bladder tissue at 0.25 mpp.

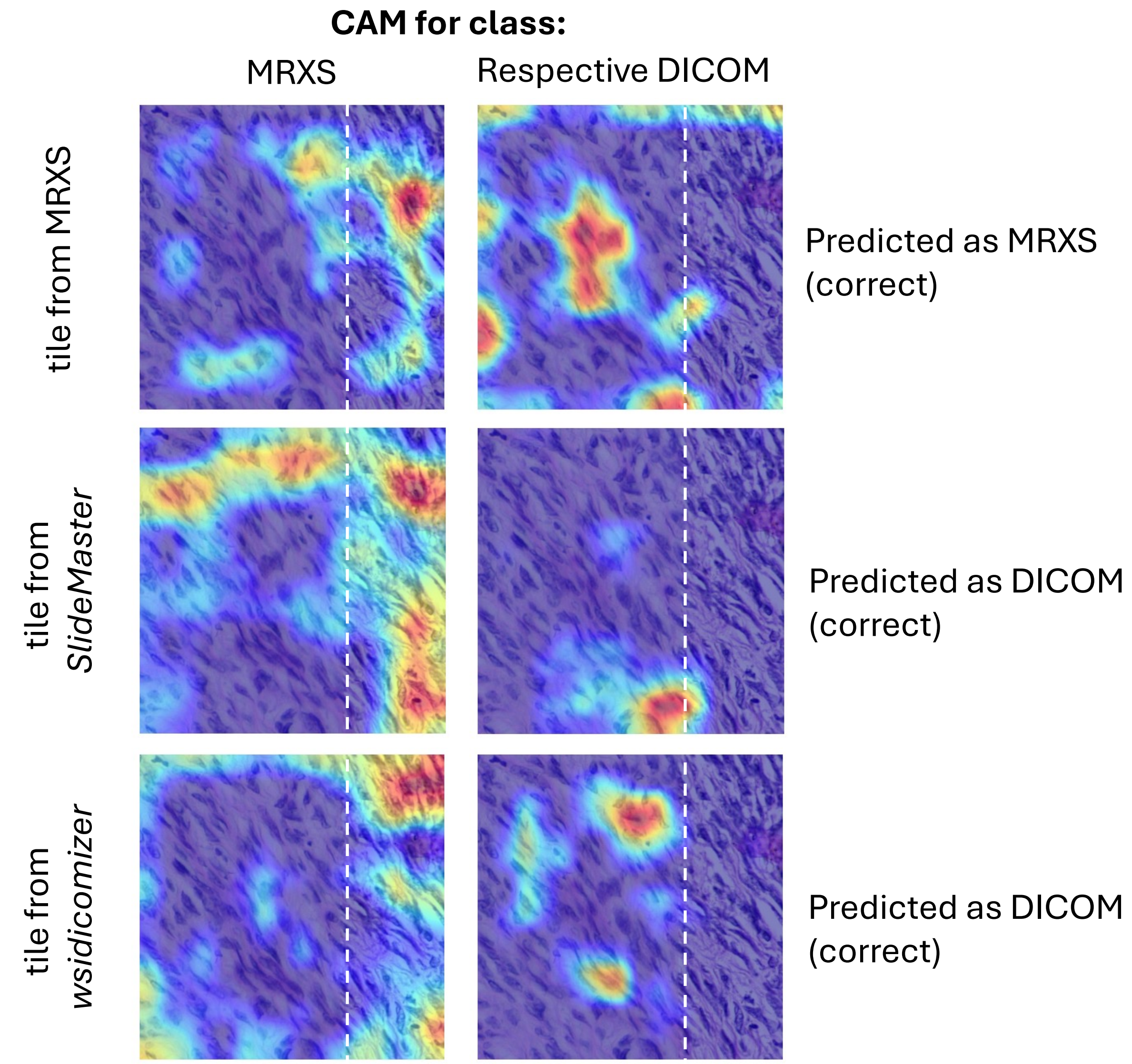

Figure S9: GradCAMs for ResNet18 predicting tile origin file format in an ovar tile. ResNet18 trained to detect file format (original vs. DICOM-converted) for two conversion tools *wsidicomizer* (Model A) and *SlideMaster* (Model B). Shown are example GradCAMs for the different classes when using as input a tile from original MRXS (top row, Model A), tile from *SlideMaster* converted slides (middle row, Model B) and a tile from *wsidicomizer* converted slides (bottom row, Model A). The dashed line signals the transition of a blurry area (left) to a sharper area (right).

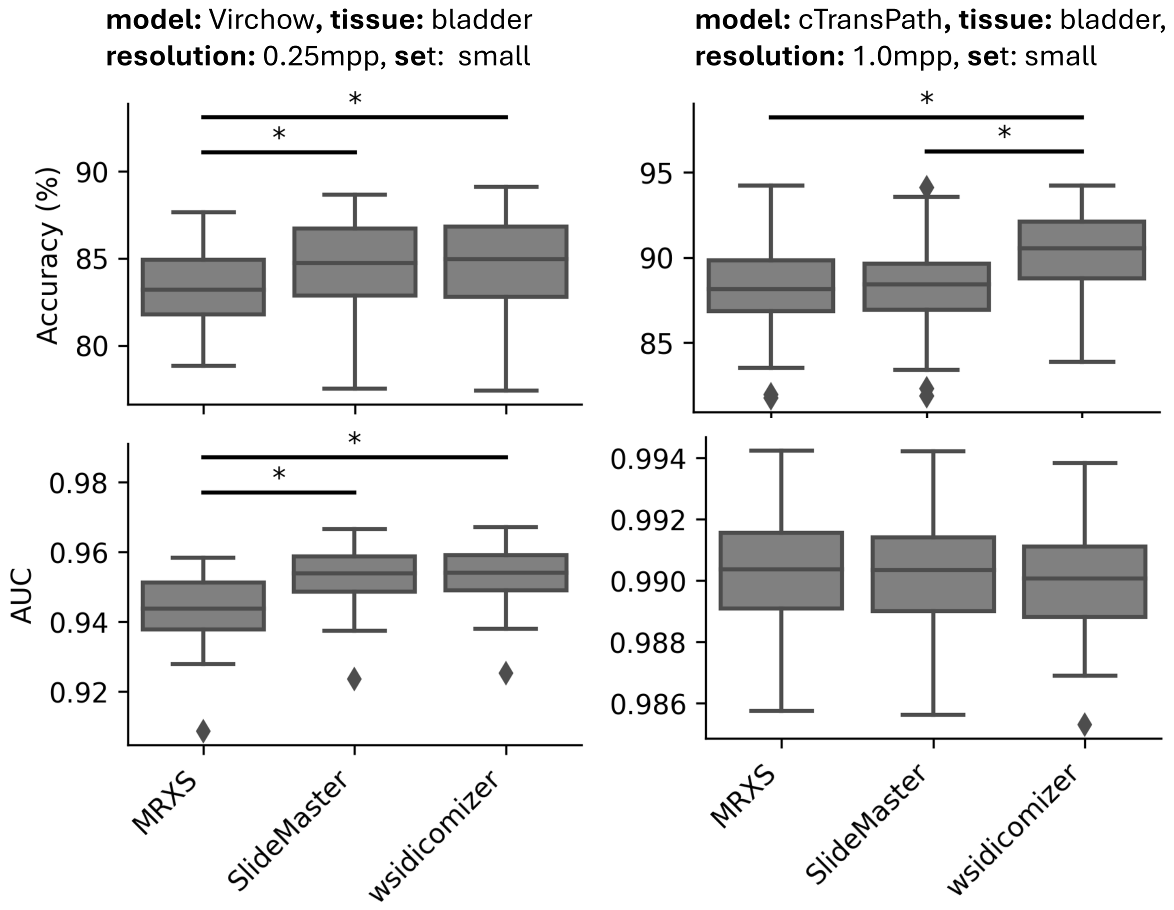

Figure S10: Possible performance increase between original and DICOM images exist during AI model inference. AI models (ResNet18, cTransPath, Virchow, and Virchow2) were trained on TCGA data and tested on local SIP data for the original and DICOM converted images for different resolutions and data set sizes. Accuracy and AUC are shown for Virchow trained with al small subset (n=17) from bladder cancer at a resolution of 0.25 mpp and cTransPath trained with a small subset (n=17) from bladder at a resolution of 1.0 mpp. Both show a case where accuracy and/or AUC could be improved, but the differences were not statistically significant.

Accuracy (%)			ResNet18			cTransPath			Virchow			Virchow2		
			mrxs	*SlideMaster*	*wsidicomizer*	mrxs	*SlideMaster*	*wsidicomizer*	mrxs	*SlideMaster*	*wsidicomizer*	mrxs	*SlideMaster*	*wsidicomizer*
**tissue**	**mpp**	**subset**												
bladder	0.25	big	77.88 ± 5.79	77.85 ± 5.88	77.9 ± 5.75	73.48 ± 3.4	72.8 ± 3.6	72.61 ± 3.5	77.12 ± 3.2	77.93 ± 3.47	77.76 ± 3.44	87.64 ± 1.73	87.3 ± 1.85	87.06 ± 1.79
		small	67.69 ± 6.7	68.52 ± 6.46	68.46 ± 6.38	84.66 ± 3.43	84.46 ± 3.5	84.5 ± 3.49	83.39 ± 2.33	84.53 ± 2.85	84.62 ± 2.86	83.92 ± 3.39	82.88 ± 3.59	82.59 ± 3.59
	1.0	big	83.61 ± 4.96	83.79 ± 5.02	83.92 ± 4.32	85.45 ± 1.7	85.34 ± 1.79	86.35 ± 1.82	83.51 ± 1.95	84.02 ± 1.92	84.01 ± 1.7	86.72 ± 1.61	87.71 ± 1.56	87.69 ± 1.5
		small	78.77 ± 6.0	78.97 ± 5.85	77.97 ± 6.08	88.34 ± 2.63	88.42 ± 2.63	90.26 ± 2.35	91.34 ± 0.82	91.1 ± 0.86	87.96 ± 1.05	85.02 ± 1.68	85.91 ± 1.56	85.85 ± 1.6
ovar	0.25	big (site A)	79.34 ± 10.16	79.68 ± 10.1	79.88 ± 10.01	92.55 ± 1.23	92.57 ± 1.2	92.58 ± 1.22	90.29 ± 2.63	89.81 ± 2.83	89.59 ± 3.02	92.22 ± 1.21	92.28 ± 1.16	92.34 ± 1.14
		small (site A	84.49 ± 7.06	84.66 ± 6.91	84.41 ± 7.21	92.58 ± 1.67	92.63 ± 1.67	92.66 ± 1.66	91.7 ± 1.78	91.39 ± 1.92	91.29 ± 2.0	92.56 ± 0.92	92.62 ± 0.91	92.66 ± 0.88
		big (site A+B)	84.8 ± 5.01	85.34 ± 4.92	85.66 ± 4.78	92.83 ± 0.66	92.86 ± 0.65	92.9 ± 0.66	92.55 ± 1.58	92.46 ± 1.75	92.48 ± 1.8	92.71 ± 1.01	92.64 ± 0.99	92.6 ± 0.98
		small (site A+B)	83.02 ± 8.15	83.01 ± 8.12	82.91 ± 8.3	93.28 ± 0.57	93.35 ± 0.53	93.41 ± 0.54	93.35 ± 0.93	93.2 ± 1.01	93.12 ± 1.08	94.94 ± 0.76	94.94 ± 0.71	94.92 ± 0.75
		big (site B)	86.48 ± 6.69	86.58 ± 6.63	86.36 ± 6.85	92.43 ± 0.94	92.5 ± 0.89	92.54 ± 0.89	91.7 ± 1.7	91.67 ± 1.71	91.73 ± 1.68	94.11 ± 0.45	94.04 ± 0.51	94.05 ± 0.51
		small (site B)	86.3 ± 6.6	85.82 ± 6.85	85.75 ± 7.14	93.24 ± 0.53	93.25 ± 0.52	93.29 ± 0.53	90.91 ± 2.26	90.54 ± 2.44	90.34 ± 2.47	93.82 ± 0.71	93.78 ± 0.74	93.81 ± 0.72
	1.0	big (site A)	84.62 ± 6.31	84.69 ± 5.93	84.68 ± 5.64	94.76 ± 1.35	94.73 ± 1.36	94.66 ± 1.35	92.76 ± 1.56	92.76 ± 1.57	92.94 ± 1.64	94.78 ± 0.43	94.73 ± 0.43	94.76 ± 0.43
		small (site A	87.17 ± 4.36	87.25 ± 4.28	85.23 ± 4.63	94.85 ± 1.32	94.82 ± 1.31	94.8 ± 1.37	93.6 ± 1.25	93.48 ± 1.27	93.41 ± 1.31	95.14 ± 0.36	95.13 ± 0.37	95.17 ± 0.41
		big (site A+B)	88.24 ± 4.39	88.18 ± 4.38	85.62 ± 5.57	95.17 ± 0.54	95.13 ± 0.54	95.14 ± 0.58	93.54 ± 1.26	93.48 ± 1.3	93.18 ± 1.48	94.53 ± 0.35	94.55 ± 0.35	94.6 ± 0.34
		small (site A+B)	89.78 ± 4.56	89.78 ± 4.62	87.11 ± 6.25	95.46 ± 0.4	95.46 ± 0.41	95.43 ± 0.42	88.24 ± 2.14	88.02 ± 2.19	87.63 ± 2.17	95.26 ± 0.43	95.22 ± 0.46	95.16 ± 0.49
		big (site B)	77.84 ± 6.93	77.72 ± 6.98	76.63 ± 6.51	94.94 ± 0.27	94.92 ± 0.27	94.88 ± 0.28	88.85 ± 4.04	88.64 ± 4.0	88.07 ± 4.58	95.03 ± 0.56	94.99 ± 0.56	94.84 ± 0.58
		small (site B)	82.65 ± 6.27	82.39 ± 6.44	81.4 ± 6.28	94.76 ± 0.45	94.74 ± 0.47	94.74 ± 0.44	88.21 ± 2.15	88.02 ± 2.13	87.48 ± 2.29	95.18 ± 0.46	95.16 ± 0.45	95.09 ± 0.47

AUC			ResNet18			cTransPath			Virchow			Virchow2		
			mrxs	*SlideMaster*	*wsidicomizer*	mrxs	*SlideMaster*	*wsidicomizer*	mrxs	*SlideMaster*	*wsidicomizer*	mrxs	*SlideMaster*	*wsidicomizer*
**tissue**	**mpp**	**subset**												
bladder	0.25	big	0.901 ± 0.035	0.899 ± 0.038	0.898 ± 0.037	0.977 ± 0.004	0.976 ± 0.004	0.976 ± 0.004	0.923 ± 0.013	0.924 ± 0.013	0.924 ± 0.013	0.947 ± 0.011	0.945 ± 0.012	0.944 ± 0.012
		small	0.839 ± 0.045	0.84 ± 0.046	0.841 ± 0.047	0.979 ± 0.004	0.979 ± 0.004	0.978 ± 0.004	0.943 ± 0.01	0.953 ± 0.008	0.953 ± 0.008	0.936 ± 0.015	0.932 ± 0.015	0.93 ± 0.016
	1.0	big	0.929 ± 0.022	0.931 ± 0.022	0.925 ± 0.022	0.992 ± 0.001	0.992 ± 0.001	0.991 ± 0.002	0.971 ± 0.007	0.973 ± 0.007	0.972 ± 0.008	0.992 ± 0.002	0.993 ± 0.002	0.992 ± 0.002
		small	0.925 ± 0.041	0.925 ± 0.04	0.92 ± 0.043	0.99 ± 0.002	0.99 ± 0.002	0.99 ± 0.002	0.985 ± 0.002	0.985 ± 0.002	0.978 ± 0.003	0.993 ± 0.002	0.993 ± 0.002	0.992 ± 0.002
ovar	0.25	big (site A)	0.955 ± 0.021	0.956 ± 0.021	0.955 ± 0.02	0.983 ± 0.003	0.983 ± 0.003	0.983 ± 0.003	0.953 ± 0.014	0.95 ± 0.017	0.948 ± 0.018	0.98 ± 0.004	0.98 ± 0.004	0.98 ± 0.004
		small (site A	0.957 ± 0.013	0.957 ± 0.013	0.956 ± 0.013	0.981 ± 0.006	0.981 ± 0.006	0.981 ± 0.006	0.959 ± 0.011	0.957 ± 0.011	0.957 ± 0.012	0.98 ± 0.005	0.98 ± 0.005	0.98 ± 0.005
		big (site A+B)	0.953 ± 0.015	0.954 ± 0.015	0.952 ± 0.015	0.978 ± 0.002	0.978 ± 0.002	0.978 ± 0.002	0.969 ± 0.009	0.968 ± 0.009	0.968 ± 0.009	0.99 ± 0.001	0.99 ± 0.001	0.99 ± 0.001
		small (site A+B)	0.96 ± 0.013	0.961 ± 0.013	0.96 ± 0.013	0.985 ± 0.002	0.985 ± 0.002	0.985 ± 0.002	0.974 ± 0.005	0.973 ± 0.005	0.973 ± 0.005	0.991 ± 0.002	0.991 ± 0.002	0.991 ± 0.002
		big (site B)	0.963 ± 0.012	0.964 ± 0.013	0.963 ± 0.014	0.973 ± 0.004	0.974 ± 0.004	0.974 ± 0.004	0.976 ± 0.005	0.975 ± 0.006	0.975 ± 0.006	0.988 ± 0.002	0.988 ± 0.002	0.989 ± 0.002
		small (site B)	0.954 ± 0.01	0.954 ± 0.011	0.954 ± 0.011	0.979 ± 0.002	0.979 ± 0.002	0.98 ± 0.002	0.972 ± 0.01	0.971 ± 0.01	0.97 ± 0.01	0.987 ± 0.004	0.987 ± 0.004	0.987 ± 0.004
	1.0	big (site A)	0.946 ± 0.023	0.944 ± 0.024	0.939 ± 0.028	0.988 ± 0.002	0.988 ± 0.002	0.987 ± 0.002	0.98 ± 0.004	0.98 ± 0.004	0.981 ± 0.004	0.988 ± 0.004	0.988 ± 0.004	0.988 ± 0.004
		small (site A	0.952 ± 0.02	0.951 ± 0.02	0.944 ± 0.024	0.991 ± 0.002	0.991 ± 0.002	0.991 ± 0.002	0.975 ± 0.004	0.975 ± 0.004	0.976 ± 0.003	0.994 ± 0.003	0.994 ± 0.003	0.994 ± 0.003
		big (site A+B)	0.964 ± 0.013	0.963 ± 0.013	0.956 ± 0.016	0.986 ± 0.002	0.986 ± 0.002	0.985 ± 0.002	0.968 ± 0.004	0.968 ± 0.004	0.968 ± 0.004	0.993 ± 0.002	0.993 ± 0.002	0.993 ± 0.002
		small (site A+B)	0.975 ± 0.019	0.974 ± 0.018	0.97 ± 0.021	0.989 ± 0.003	0.988 ± 0.003	0.988 ± 0.003	0.978 ± 0.003	0.977 ± 0.003	0.976 ± 0.003	0.992 ± 0.002	0.992 ± 0.002	0.992 ± 0.002
		big (site B)	0.883 ± 0.054	0.883 ± 0.054	0.873 ± 0.054	0.984 ± 0.005	0.983 ± 0.005	0.983 ± 0.005	0.974 ± 0.004	0.974 ± 0.004	0.974 ± 0.004	0.996 ± 0.002	0.996 ± 0.002	0.996 ± 0.002
		small (site B)	0.928 ± 0.034	0.926 ± 0.034	0.913 ± 0.041	0.991 ± 0.002	0.991 ± 0.002	0.99 ± 0.002	0.97 ± 0.005	0.969 ± 0.005	0.969 ± 0.005	0.995 ± 0.002	0.995 ± 0.002	0.995 ± 0.002

Significant differences in group present: before Bonferroni correction (blue); after Bonferroni correction (orange)

Table S1: Performance of ResNet18 and Foundation Model-Based Feature Extractors for Carcinoma Detection. ResNet18 models and models leveraging foundation model-based features (cTransPath, Virchow, and Virchow2) were trained on TCGA data for carcinoma detection in bladder and ovarian tissues. The datasets varied in resolution (0.25 mpp and 1.0 mpp) and training sizes (small and big). For ovarian tissue, additional runs were performed for two individual tissue source sites as well as their combined dataset. The models were evaluated on test data from SIP using three formats: original MRXS slides, tiles derived from *SlideMaster*-converted slides, and tiles derived from *wsidicomizer*-converted slides. Average accuracy (top) and AUC (bottom) are presented along with their respective standard deviations (±). Cases with significant differences between the three test formats (ANOVA/Kruskal-Wallis <0.05) are highlighted in blue, while values remaining significant after p-value correction (Bonferroni < 0.05) are marked in orange.

Accuracy (%)			ResNet18			cTransPath			Virchow			Virchow2		
		trainfiletype →	mrxs	*SlideMaster*	*wsidicomizer*	mrxs	*SlideMaster*	*wsidicomizer*	mrxs	*SlideMaster*	*wsidicomizer*	mrxs	*SlideMaster*	*wsidicomizer*
tissue	mpp	size												
bladder	0.25	small	70.35 ± 11.67	62.28 ± 8.94	64.82 ± 10.57	89.39 ± 2.25	88.49 ± 1.94	89.68 ± 1.88	95.62 ± 0.44	95.82 ± 0.38	95.82 ± 0.44	96.29 ± 0.49	96.48 ± 0.39	96.37 ± 0.44
	1.0	small	82.79 ± 6.85	82.94 ± 8.47	81.75 ± 8.02	98.81 ± 0.38	98.69 ± 0.33	98.78 ± 0.35	97.65 ± 0.79	97.75 ± 0.74	98.08 ± 0.92	97.32 ± 0.55	97.19 ± 0.55	96.7 ± 0.86
ovar	0.25	big	72.68 ± 8.73	72.69 ± 8.5	71.79 ± 9.42	88.14 ± 1.95	87.79 ± 2.14	87.86 ± 1.96	59.81 ± 3.81	59.79 ± 4.06	62.43 ± 5.84	85.32 ± 0.99	84.73 ± 1.1	84.95 ± 1.15
		small	69.8 ± 13.2	68.93 ± 11.97	68.55 ± 11.85	88.29 ± 2.94	88.23 ± 3.19	88.08 ± 2.91	61.77 ± 6.56	62.35 ± 7.04	62.86 ± 6.89	84.52 ± 2.23	84.33 ± 2.35	84.35 ± 2.53
	1.0	big	70.12 ± 6.42	69.13 ± 7.74	69.99 ± 6.4	89.98 ± 0.72	89.87 ± 0.83	89.88 ± 0.8	86.18 ± 0.95	86.24 ± 1.03	85.71 ± 1.08	86.3 ± 1.74	86.42 ± 1.97	86.21 ± 1.85
		small	66.36 ± 9.3	67.27 ± 10.64	68.86 ± 8.6	89.69 ± 1.29	89.67 ± 1.26	89.66 ± 1.33	85.06 ± 1.54	84.91 ± 1.72	84.37 ± 1.64	83.99 ± 3.4	83.58 ± 3.51	83.09 ± 3.28

AUC			ResNet18			cTransPath			Virchow			Virchow2		
		trainfiletype →	mrxs	*SlideMaster*	*wsidicomizer*	mrxs	*SlideMaster*	*wsidicomizer*	mrxs	*SlideMaster*	*wsidicomizer*	mrxs	*SlideMaster*	*wsidicomizer*
tissue	mpp	size												
bladder	0.25	small	0.947 ± 2.183	0.943 ± 1.923	0.942 ± 2.207	0.978 ± 0.371	0.978 ± 0.336	0.979 ± 0.351	0.994 ± 0.053	0.994 ± 0.059	0.994 ± 0.06	0.996 ± 0.05	0.996 ± 0.065	0.996 ± 0.06
	1.0	small	0.989 ± 0.503	0.989 ± 0.661	0.99 ± 0.401	0.999 ± 0.031	0.999 ± 0.03	0.999 ± 0.023	0.998 ± 0.077	0.999 ± 0.065	0.999 ± 0.046	0.999 ± 0.056	0.999 ± 0.059	0.999 ± 0.066
ovar	0.25	big	0.938 ± 1.875	0.939 ± 1.826	0.935 ± 2.045	0.958 ± 0.788	0.957 ± 0.854	0.957 ± 0.841	0.865 ± 2.55	0.863 ± 2.415	0.864 ± 2.723	0.933 ± 1.048	0.93 ± 1.219	0.93 ± 1.265
		small	0.917 ± 5.21	0.914 ± 4.813	0.922 ± 3.612	0.962 ± 1.649	0.962 ± 1.728	0.961 ± 1.681	0.859 ± 3.753	0.858 ± 4.078	0.858 ± 3.469	0.935 ± 1.648	0.934 ± 1.703	0.934 ± 1.811
	1.0	big	0.927 ± 1.331	0.923 ± 2.061	0.918 ± 2.135	0.965 ± 0.404	0.964 ± 0.47	0.964 ± 0.444	0.936 ± 0.73	0.936 ± 0.748	0.933 ± 0.779	0.963 ± 0.367	0.962 ± 0.384	0.962 ± 0.356
		small	0.909 ± 2.966	0.904 ± 3.722	0.909 ± 2.729	0.964 ± 0.629	0.964 ± 0.631	0.963 ± 0.701	0.929 ± 1.321	0.927 ± 1.327	0.925 ± 1.383	0.959 ± 0.63	0.958 ± 0.636	0.957 ± 0.807

Significant differences in group present: before Bonferroni correction (blue); after Bonferroni correction (orange)

Table S2: Comparison of performance of training on DICOM-converted images against original MRXS. ResNet18 models and models leveraging foundation model-based features (cTransPath, Virchow, and Virchow2) were trained on SIP data for carcinoma detection in bladder and ovarian tissues. The training was repeated on the same tile-sets for original, *SlideMaster* converted and *wsidicomizer* converted images. The datasets varied in resolution (0.25 mpp and 1.0 mpp) and in ovar also training sizes (small and big). For The models were evaluated on test data from the TCGA Average accuracy (top) and AUC (bottom) are presented along with their respective standard deviations (±). Cases with significant differences between the three test formats (ANOVA/Kruskal-Wallis <0.05) are highlighted in blue, while values remaining significant after p-value correction (Bonferroni < 0.05) are marked in orange.

Detailed Methods

To evaluate the impact of DICOM conversion on the resulting images and AI models tiles from different datasets and tissues were extracted with their respective counterparts from the same slide after DICOM conversion. Extracted tiles were analyzed for structural similarity and further used for several AI training scenarios.

Datasets

Ovarian tissue datasets consisted of 72 whole slide images (WSIs) from 1 patient each acquired via the TCGA-OV database. Additionally, 38 WSIs from 1 patient each were locally sourced from the SIP cohort and scanned using the PanoramicScanII (version 2.1.1.100094 & 3.0.1.123298). For bladder tissue, 107 WSIs from 1 patient each were obtained from the TCGA-MIBC, complemented by 17 WSIs from 1 patient each acquired locally from the SIP cohort, also scanned with the PanoramicScanII (version 3.0.1.123298). Finally, prostate tissue data included 40 WSIs from 1 patient locally acquired from the SIP cohort using the P1000 scanner (version 2.2.1.179491).
All slides from TCGA were provided in Leica Aperio SVS format and nominally at 40x magnification. Similarly, local SIP data were scanned at 40x magnification but provided in MRXS format. For both ovarian and bladder datasets from TCGA, cancerous regions were annotated by a pathologist using the QuPath brush function (version 0.4.3). Additionally, circle annotations marking healthy and cancerous tissue were created for SIP cohort slides using the Sysmex Caseviewer software.

Processing

To enable feasible AI applications, smaller image tiles were extracted from the WSIs at resolutions of 299×299 pixels (bladder, prostate) and 512×512 pixels (ovarian). For the bladder and ovarian datasets, 40 tiles were randomly sampled per patient, with 20 tiles from each annotated class (healthy and cancerous). The exact number of tiles per patient and class varied slightly due to differences in annotation methods and the distribution of tissue types within each slide. For the prostate dataset, 40 tiles were extracted at random without class-specific annotations.
Tile extraction was performed using OpenSlide (version 1.4.1) at two resolutions: 0.25 mpp and 1.0 mpp. Tiles at the higher magnification (0.25 mpp) were derived from the same tissue regions as their lower magnification (1.0 mpp) counterparts, representing a more zoomed-in view of the same area.

DICOM conversion

Finally, all MRXS slides were converted to DICOM format using the Sysmex *SlideMaster* software (version 2.7.0.206362) with default parameters (JPEG (ISO_109181), Q=80, chroma subsampling = 4:2:0). Tiles were extracted from the converted WSIs at the same resolutions and coordinates as the previously extracted tiles using OpenSlide. However, aligning the coordinate systems between MRXS and DICOM slides was necessary due to irregular coordinate offsets introduced by the *SlideMaster* conversion.

To achieve this, a rough alignment was first performed by shifting coordinates until two extracted tiles visually matched. This was followed by fine-tuning: the coordinates were adjusted pixel by pixel within a grid surrounding the initial alignment. At each step, the structural similarity index (SSIM) between the tiles was calculated using the scikit-image function *structural_similarity* (version 0.24.0), and the shift yielding the highest SSIM was selected for final alignment.

In addition, a second and third DICOM dataset were created by converting MRXS slides using the open-source tool *wsidicomizer* with default parameters (JPEG (ISO_109181), Q=90, chroma subsampling = 4:2:2) and with settings adjusted to match *SlideMaster* (JPEG (ISO_109181), Q=80, chroma subsampling = 4:2:0). Tiles were extracted at the same coordinates and resolutions as the original datasets. For this dataset, no manual alignment was required. However, the slides had to be accessed via the *WSIDicom* library (version 0.10.0), as OpenSlide can not process this file structure.

Similarity Measures

To determine changes introduced by conversion, structural similarity indices (SSIM) were determined between MRXS images and their respective counterparts from the three DICOM datasets. For this scikit-image *structural_similarity* (version 0.24.0) was used via multichannel analysis.

AI Models

For all further analyses only the *wsidicomizer* DICOM dataset that was converted via default settings was used. Four approaches were utilized for AI analysis in this study. First, a convolutional neural network (CNN) architecture, ResNet18, was implemented using the timm library, with pretrained ImageNet weights. Added to this model was a custom multi-layer perceptron (MLP) head consisting of 512 neurons, a dropout layer of 0.5, and an output layer of size two for classification. All layers, including the convolutional layers, were trained. Training parameters included optimizer, loss function.

Additionally, three foundation models, cTransPath, Virchow, and Virchow2, were used as transformer-based feature extractors. These models generated feature vectors of sizes 768, 2560, and 2560, respectively. The extracted feature vectors were then fed into a custom MLP head with an initial layer matching the size of the respective feature vector, followed by a dropout layer (0.5), and a final output layer of size two for classification.

File Format Detections

For all three datasets, ovarian, bladder, and prostate, training was performed for each of the models. Only MRXS-based tiles and their exact DICOM counterparts were used, with the models trained to distinguish between the two file formats. The data was split into 13 training patients, 2 validation patients, and a holdout test set of 2 patients. This process was performed separately for both versions of the DICOM format.

For each resolution, tissue type, and conversion tool, the models were trained for 20 epochs. Performance metrics, including accuracy and area under the curve (AUC), were calculated for each training iteration. To ensure robustness, the training process was repeated 50 times with different train-test splits. These experiments were conducted for both the 0.25 mpp and 1.0 mpp resolutions.
Finally, Grad-CAMs were generated for a subset of test tiles using the ResNet18 models to provide visual explanations of the model's prediction process.

Cancer Detection

Next, for the ovarian and bladder datasets, all models were trained as cancer detection models at both 0.25 mpp and 1.0 mpp. The training involved deriving multiple scenarios from the TCGA datasets. For the bladder dataset, models were trained using a large (all 107 slides) and a small (17 slides) dataset. For the ovarian dataset, training was conducted using different tissue source sites from the TCGA, which we will refer to as A and B, as well as their combination. This was repeated for both a large (all available slides: A 35, B 37, A+B 72) and a small size (17 slides). All models were trained for 20 epochs, and this process was repeated 50 times with varying train-validation splits to enhance robustness. The MRXS and two DICOM datasets were used for testing the respective tissue models independently, allowing a comparison between MRXS and the two DICOM formats. This led to a total of 9500 trained models across 64 individual test scenarios. For these scenarios, statistical tests were conducted to determine the significance of test performance differences between the MRXS and DICOM test sets. If the assumptions were met, the appropriate statistical method (ANOVA) was utilized; otherwise, a non-parametric method (Kruskal-Wallis) was used. Finally, p-values were corrected via the Bonferroni correction to account for multiple comparisons. Pairwise significance was determined via the appropriate test (Tukey's HSD / Mann-Whitney U-test).

File type dependent AI training

Models were always trained on SIP data (original MRXS or converted DICOM) and tested on TCGA data. For each tissue, all available UKF-based slides were used (bladder: 17, ovar:38). For the ovarian dataset training was performed additionally with a small dataset size (17 WSIs). As we have two resolutions and four architecture this results in 24 tested scenarios ((1 bladder cancer datasets + 2 ovarian cancer datasets)*2 resolutions *4 models = 24).
